# Allometric scaling of biomass with nitrogen and phosphorus above- and below-ground in herbaceous plants varies along water-salinity gradients

**DOI:** 10.1093/aobpla/plab030

**Published:** 2021-06-09

**Authors:** An Na Liu, Yang Zhang, Zhu Feng Hou, Guang Hui Lü

**Affiliations:** 1 Institute of Arid Ecology and Environment, Xinjiang University, Urumqi 830046, China; 2 Key Laboratory of Oasis Ecology, Education Ministry, Urumqi 830046, China; 3 Institute of Resources and Environment Sciences, Xinjiang University, Urumqi 8300462, China; 4 College of Science, Shihezi University, Shehezi 832003, China

**Keywords:** Allometric scaling, biomass allocation, ecological stoichiometry, herbaceous plants, water-salinity gradient

## Abstract

Biomass allocation affects the ability of plants to acquire resources and nutrients; a limited allocation of nutrients, such as nitrogen and phosphorus, affects ecological processes. However, little research has been conducted on how plant allocation patterns change and on the trade-offs involved in allocation strategies when microhabitat gradients exist. We selected a 3.6 km transect in the Ebinur Lake Wetland Natural Reserve of Xinjiang, China, to investigate the relationships between plant traits (biomass and N and P concentrations) of herbaceous plants and environmental factors (soil moisture, salinity and nutrient content), and to determine the allometric scaling of biomass and stoichiometric traits between the above- and below-ground plant parts. The results show that the biomass and stoichiometric traits of plants reflected both the change of micro-environment and the natural characteristics of plants. With a decrease of the soil water availability and salinity, above- and below-ground N and P concentrations decrease gradually; scaling relationships exist between above- and below-ground plant parts, for biomass and N and P concentrations. Biomass allocation is influenced by soil nutrient ratios, and the allocation strategy tended to be conserved for N and variable for P. Second, the scaling relationships also show interspecific differences; all scaling exponents of *Suaeda prostrata* are larger than for other species and indicate a ‘tolerance’ strategy, while other species tend to increase the below-ground biomass and N and P concentrations, i.e. a ‘capture’ strategy.

## Teaser

In the ecological research in the Ebinur Lake Wetland Natural Reserve of Xinjiang, China, we found that above- and below-ground N and P concentrations decrease with a decrease in soil water availability and salinity; and that biomass allocation is influenced by soil nutrient ratios. Our results show the different allometric scaling of biomass and stoichiometric traits between the above- and below-ground plant parts, and interspecific allocation strategies of ‘tolerance’ (increase the above-ground biomass) and ‘capture’ (increase the below-ground biomass).

## Introduction

The above- and below-ground parts of herbaceous plants depend on each other directly, which is closely related to capturing and using light, nutrients and water resources. The appropriate allocation of resources above- and below-ground is an important strategy plants use to adapt to environmental heterogeneity, the study of which focuses on the allocation of biomass (Niklas and Enquist 2001, 2002), morphological plasticity (Grime and Mackey 2002) and functional traits (Reich 2003). Significant differences have been observed in allocation strategies among species and habitats (Poorter and Nagel 2000). For example, plants typically allocate limited resources differently among organs; that is, plants will compensate for the organs that capture given resources when a required resource is in short supply. The plant may allocate more resources below-ground to compensate for a shortage of underground resources (Brouwer 1963; Tilman 1991; Reynolds and Chen 1996; Craine *et al*. 2002). According to the ‘optimal partitioning theory’ or ‘functional equilibrium’ (Poorter *et al*. 2012; Gedroc *et al*. 1996), a trade-off exists between various allocation strategies, and plants will allocate limited resources in the optimal way to achieve functional equilibrium under environmental stress. The allocation of resources to root (Gleeson and Tilman 1990; Shipley and Meziane 2002; Robinson *et al*. 2010) and leaf (Kerkhoff *et al*. 2006; Xia and Wan 2008) has been well studied. Water limitation is an important environmental stress in arid areas (Mokany *et al*. 2006; Snyman 2009). With decreasing precipitation, there is a shift from the allocation of more above-ground towards more below-ground biomass (Santiago *et al*. 2005; He *et al*. 2008). And the drought often accompanies by increased salinization, with the increase of soil salinity and pH (Elamin and Hussein 2000), and then inhibited plant growth. In the study of two dominant plants in Inner Mongolia grassland, there was interspecific different of the ecological strategy in the face of water stress (Chen *et al*. 2013a).

The covariant relationship among organs of a plant can be transformed into a scaling relationship of one trait variable to another to test this hypothesis (Elser 2006; Schreeg *et al*. 2014; Yang *et al*. 2014; Zhu *et al*. 2015). This scaling relationship has been widely used to explain the patterns of metabolic and physiological traits (Brown *et al*. 2004; Price *et al*. 2012; Xiang *et al*. 2013). In addition to biomass, nutrient allocation among organs also shows a specific scaling relationship (Bazzaz and Grace 1997; Sardans and Peñuelas 2013). Harris (1992) considered that nitrogen would promote the growth of the crown while phosphorus facilitated greater root growth based on a study of the differential responses of the root and crown to N or P. Previous studies have suggested that plants tend to compete for nutrients in infertile conditions, rather than for light resources (Tilman 1988); specifically, the species with more below- than above-ground biomass have an advantage in competing for nutrients. This suggested that allometric relationships constrain how plants allocate both above- and below-ground biomass and nutrients, and that nutrient limitation may affect the biomass allocation strategy of an individual (Andrews *et al*. 1999; Müller *et al*. 2000; Güsewell 2004; Yang *et al*. 2009).

The allocation of nutrients reflects the interaction between plants and the environment. The allometric allocation of plants may depend on the specific species involved (McCarthy and Enquist 2007), functional groups (Li *et al*. 2010) and different types of environmental stress (Craine 2005; Liu 2010). Specifically, the biotic factors of species, functional group and environmental factors of climate and soil properties play significant roles in plant functional traits (Reich *et al*. 1997; Westoby 2004; Chen *et al*. 2013b; Yao *et al*. 2015); therefore, we expected that the interspecific allocation difference will change with changes in environment factors, and that these two will jointly determine the unique biogeographic pattern of vegetation. At present, allocation patterns across environmental gradients are more concentrated on large-scale climatic change (temperature and precipitation). From the large-scale perspective, the proportion of the above-ground biomass in mesic sites is greater than that in dry sites (Orians and Solbrig 1977; Mooney *et al*. 1978), and the concentration of N in leaves is greater in dry than in mesic environments (Wright *et al*. 2001, 2002; Maherali and DeLucia 2001; Han *et al*. 2011). The scaling exponents of N or P between the twig, stem, and leaf decreased from >1 at low latitude to <1 in high latitude, which may be controlled by temperature (Yan *et al*. 2016). Herbaceous plants in deserts are influenced by the physical and chemical properties of soil nutrients as well as by salt and water content (Qian *et al*. 2004) except for macro-environmental factors. A simulation study found that the soil moisture regime directly affects the response of plants to the nitrogen input (Asner *et al*. 2001). Soil with a high salinity will restrict the availability of the soil nutrients by disturbing the soil N and P cycle (Schlesinger 1997). At a small scale, climate and soil texture have similar effects, so soil heterogeneity is the main factor that affects the above- and below- ground biomass and stoichiometry traits. However, the effects of these micro-environmental factors on the allometric pattern of herbaceous plants in arid and semi-arid areas remain unclear.

In the Ebinur Lake Wetland Natural Reserve of Xinjiang, China, we systematically conducted an investigation of the above- and below-ground biomass and nutrient allocation of herbaceous plants across the water-salinity gradient on the vertical direction from the river bank. We speculated that the distribution patterns of plant biomass, as well as the N and P concentrations, are different and will vary with the micro-environmental factors of soil properties. Specifically, we proposed the following three hypotheses: (i) the biomass and nutrients of herbaceous plants vary with the water-salinity gradient; (ii) different scaling relationships of biomass and nutrients exist between the above- and below-ground of plants, with the scaling relationships varying along the water-salinity gradient; (iii) a trade-off exists between the distribution of the biomass and nutrients, with the distribution patterns varying across species and functional groups.

## Materials and Methods

### Study area

The study area lies within Ebinur Lake Wetland Natural Reserve (44°31′05″N–45°09′35″N, 82°33′47″E–83°53′21″E), and has a arid temperate continental climate with a mean annual temperature, precipitation and evaporation of 5 °C, 105.17 mm and 1662 mm, respectively. The soil is a typical zonal desert soil with intrazonal soils including solonchak, meadow soil and swampy soil. The dominant species include xeric and super-xeric desert plants, which are widely but sparsely distributed; an open area was selected for the transect (Zhang *et al*. 2015).

### Sampling design

**Figure 1.**
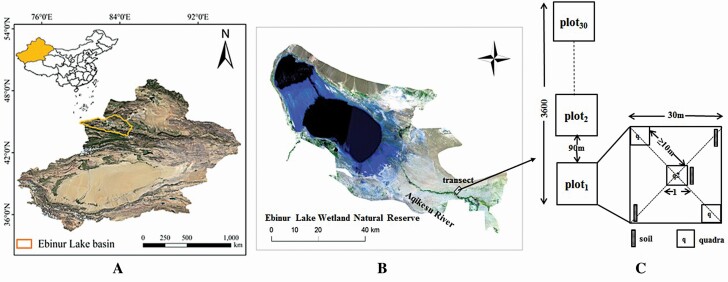
Sampling sites and plots. (A) Aerial photo of Ebinur Lake and the study site location; (B) a 3.6 km transect along the vertical direction of the Aqikesu River in the Ebinur Lake Wetland Natural Reserve; (C) a total of thirty 30 m × 30 m plots were set up at 90 m intervals in the transect, and three 1 m × 1 m quadrats were placed on the diagonal of each plot.

In July and August 2016, a north-south transect was established extending 3600 m along the vertical direction of the Aqikesu River in the desert, in which thirty 30 m × 30 m plots were established at 90 m intervals. Three 1 m × 1 m quadrats were placed in a diagonal line within each plot for the analysis of the herbaceous plant community; these were spaced more than 10 m apart (Fig. 1). In the transect and its surrounding, the trees and shrubs, including *Populus euphratica* and *Haloxylon ammodendron*, were not higher than 3 m. Previous research from the savanna and arid areas of western China has indicated that the trees and shrubs had little effect on the herbs at distances more than 1.5 times their height (Belsky *et al*. 1993; Zhang *et al*. 2007). The quadrats were set up far away (more than 7 m) from trees and shrubs so that their influence on herbs could be excluded. In addition, the variation of the soil has no significant effects on trees and shrubs, but does have significant effects on annual or perennial herbs (Li *et al*. 2004). Therefore, trees and shrubs were excluded and only select herbs were selected the object of the present study.

All herb individuals were identified to the species level; then, the cover (%), height and number of individuals were measured within each quadrat for each species. Based on the survey results, we selected the dominant or common species for sampling. Specifically, five whole plants of each selected species were excavated in each plot. The above- and below-ground tissues of each plant were detached carefully, cleaned to remove the foreign materials, and placed into separate paper envelopes. All the plant samples were transported to the laboratory for processing.

We excavated three soil profiles along the diagonal line of each quadrat, and then collected soil samples at depths of 0–10 cm, 10–20 cm and 20–30 cm. The soil samples from a single plot at the same depth were mixed into a sealed bag and numbered prior to determination of soil moisture content and salinity. After removing the topsoil (2 cm), three different soils (0–20 cm) from the stem of each plant to the outer crown were collected and mixed evenly. These samples were numbered and sealed in bags, and returned to the laboratory to determine the soil N and P concentrations.

### Plant materials

In this study, 12 herbaceous species were collected from eight genera and four families, in which 75 % of species were Chenopodiaceae distributed in desert and saline-alkali areas. Seven species were annual herbs, and five were perennial herbs. The species richness in quadrats ranged from 1 to 6, and none of the species occurred in all the plots of the transect. We selected four dominant herbaceous species with frequency more than 20 %. *Suaeda prostrate* (*S. prostrate*) occurred in 71.1 % of quadrats, and the frequency of other dominant species, such as *Halogeton glomeratus* (*H. glomeratus*)*, Suaeda salsa* (*S. salsa*) and *Phragmites australis* (*P. australis*), were 34.4, 27.8 and 21.1 %, respectively. *Suaeda prostrata*, *Halogeton glomeratus* and *Suaeda salsa* are annual herbs from the Chenopodiaceae, whereas *Phragmites australis* is a perennial herb of Gramineae.

### Measurements

Plant samples were dried at 65 °C to constant weight and the biomass of each sample was measured; then, samples were powdered to sieve through a 2-mm mesh using a pulverizer. The total N content was determined by Kjeldahl apparatus (K-370, Buchi Labortechnik AG, Falwil, Switzerland), and the total phosphorus content was analysed by molydate/ascorbic acid turbidimetry after H_2_SO_4_–H_2_O_2_ digestion.

Soil samples were air-dried and ground through a 100-mesh sieve. The soil total N and P content were determined by the same method after H_2_SO_4_–HClO_4_ digestion (Bao 2000). The soil moisture content was measured by the drying-weigh method. The soil conductivity was measured in distilled H_2_O at a ratio of 1:5, and the leaching solution was dried and the residue was weighed to represent the total amount of water-soluble salt. Soil salinity was derived from the standard curve of soil conductivity and the total amount of water-soluble salt (*r*^2^ = 0.90).

### Statistical analysis

Allometric equations were used to analyse the scaling relationship between above- and below-ground variables (biomass and N and P concentrations), which was expressed by *Y* = *βX*^*α*^ or log(*Y*) = log(*β*) + *α*log(*X*), where *X* and *Y* represent below- and above-ground variables, respectively, and *α* is the regression slope (scaling exponent) and *β* is the regression intercept. When the 95 % confidence interval contained 1, *α* indicated the scaling relationship between *Y* and *X* as isometric; otherwise it was assigned as allometric (Niklas 1994).

We analysed the scaling relationship of plant biomass and stoichiometric traits from three levels as follows: (i) all plots were divided into three groups (high, medium and low water-salinity) based on soil moisture and salinity using *K*-means clustering; (ii) all original data were divided into two functional groups of plants (annual and perennial); (iii) data were analysed according to the four dominant species with relatively high frequency (*S. prostrata*, *S. salsa*, *H. glomeratus* and *P. australis*).

After the log-transformation, model II regression (i.e. reduced major axis, RMA) was applied to estimate the regression parameters (Sokal and Rohlf 1981, Warton *et al*. 2012), and a one-sample test was used to test for a significant difference between the regression slope and 1. If the regression relationship was not significant (i.e. *P* > 0.01), we set 0 to *α*; that is, the organism was considered to have a strictly allometric relationship **[see****Supporting Information—Appendix A1****–****A4****]**. A Bayesian model was used to estimate the parameters and to determine whether the estimation range include 0 (Rue 2009).

To test the response of above- and below-ground stoichiometric traits to the water-salinity gradient, we analysed the variation in the traits of each specific plant for the effects of water-salinity and soil nutrients using the analysis of covariance, with the gradient as an independent variable and soil nutrients as a covariable. All statistical analyses were performed using R 3.4.2 with smatr, INLA and HH packages (R Core Team 2014).

## Results

### Water-salinity gradients in the study area

**Figure 2.**
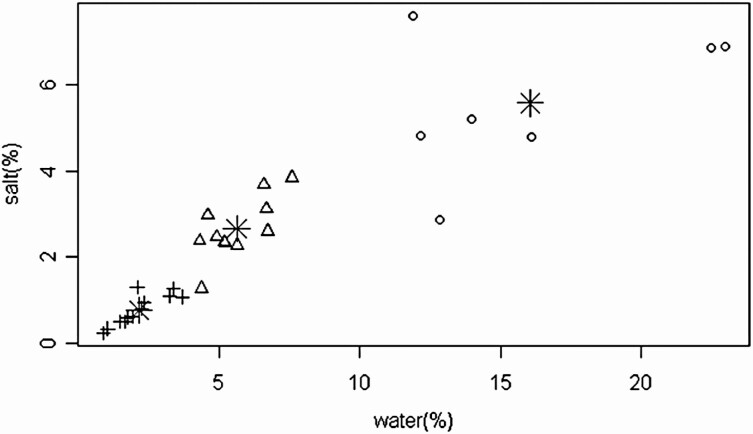
Relationship between soil water and salinity-water-salinity gradient distribution. The circles, triangles and crosses represent high (HWS), medium (MWS) and low (LWS) water-salinity plots, respectively. The symbol represents the central points of the three gradients.

The clustering results showed that the variation of the soil moisture and salinity have the same trend (*r*^2^ = 0.82, *P* < 0.001), with the highest moisture and salinity of soil near the river and the lowest near the desert. A spatial distribution pattern was observed for high (HWS), medium (HWS) and low water-salinity (LWS; *P* < 0.001). Thus, the 30 plots can be divided into three groups (Fig. 2), and there were different soil properties among groups (Table 1). Those with HWS included plots 1, 2, 3, 4, 5, 6, 7 and 8; MWS included plots 9, 10, 11, 12, 13, 14, 15, 16, 17, 18 and 19; and LWS included the other plots.

**Table 1.** Soil moisture (%), total salt content (g/kg), total nitrogen (%) and total phosphorus (%) in three water-salinity gradients.

Gradient	Soil moisture (%)	Total soil salinity (g/kg)	Total nitrogen (%)	Total phosphorus (%)
HWS	16.06 ± 4.76^c^	5.58 ± 1.64^c^	0.25 ± 0.04^a^	0.43 ± 0.06^b^
MWS	5.63 ± 1.11^b^	2.67 ± 0.72^b^	0.32 ± 0.07^b^	0.45 ± 0.07^b^
LWS	2.16 ± 0.90^a^	2.12 ± 0.85^a^	0.19 ± 0.04^a^	0.33 ± 0.05^a^

Letters represent significant differences at the water-salinity gradient (*P* < 0.05).

### The stoichiometric relationships of soil and plants on a water-salinity gradient

We analysed the effects of water-salinity gradients, species and functional groups on the above- and below-ground biomass along with the N and P concentrations. The results suggested that (Table 2) water-salinity gradients had extremely significant effects on all the above- and below-ground traits (*P* < 0.001); the effects of the species on above-ground traits and below-ground biomass were very significant (*P* < 0.001), and on N, the P concentration of the below-ground tissues was also significant (*P* < 0.05); the functional groups had no significant influence on all traits. The interaction between the gradient and species had very significant effects on the above-ground biomass and below-ground N content (*P* < 0.05).

**Table 2.** Main effects and interaction effects of water-salinity gradients, species and functional groups on above- and below-ground biomass, as well as the N and P concentrations. ***P < 0.001; **P < 0.01; *P < 0.05.

	Above-ground			Below-ground		
	Biomass	N	P	Biomass	N	P
Gradient	94.45***	116.92***	10.82***	60.56***	41.85***	16.70***
Species	8.46***	19.98***	5.91***	12.05***	1.99*	2.25*
Functional group	—	—	—	—	—	—
Gradient × Species	6.13***	3.85**	2.25*	2.41*	3.99***	1.86
Gradient × Functional group	—	—	—	—	—	—

**Figure 3.**
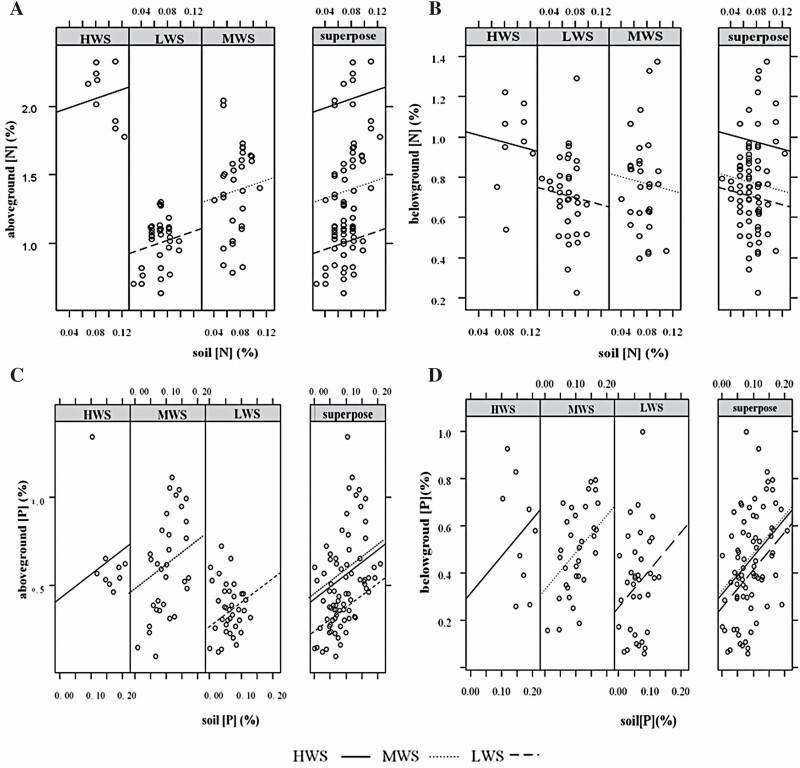
Effects of soil N and P concentrations on the above- and below-ground biomass and N, P concentrations of *S. prostrata* in water-salinity gradients. Solid, dotted and dashed lines represent high (HWS), medium (MWS) and low (LWS) water-salinity plots, respectively.

According to the results of the analysis of variance, we selected the only species (*S. prostrata*) that occurred across all three gradients to analyse the effects of the water-salinity gradient along with the soil N and P concentrations on the variation of above- and below-ground N and P concentrations using the analysis of covariance. The results showed that the regression slopes of soil with plant N and P content were the same and the intercepts were different within three gradients, so the variation trend was consistent (Fig. 3). The above-ground N concentration increased significantly (*P* < 0.001), and below-ground N decreased with increasing soil N content. The intercept of HWS was the largest and LWS was the smallest. The interaction effect of the soil and gradient was insignificant, which meant that the regression relationship between soil and plants did not depend on the gradient (Table 3). The above- and below-ground P concentrations increased significantly (*P* < 0.001) with the increasing soil P content, and the intercepts could be classified as MWS > HWS > LWS. The interaction effect between the soil and gradient was significant, and the regression relationship of the soil and above-ground P content was most strongly influenced by the gradient (*P* < 0.001).

**Table 3.** The main and interaction effects of the soil nutrient concentration and water-salinity gradient on the above- and below-ground biomass as well as N and P concentrations.

N	Above-ground		Below-ground		P	Above-ground		Below-ground	
	*F*	*P*	*F*	*P*		*F*	*P*	*F*	*P*
Soil	36.50	<0.001	0.85	0.36		24.15	<0.001	19.26	<0.001
Gradient	50.81	<0.001	4.50	<0.05		5.63	<0.01	0.95	0.39
Soil × gradient	1.87	0.16	0.70	0.50		7.94	<0.001	3.75	<0.05

### The scaling relationship of biomass with N and P above- and below-ground

#### Scaling exponents across the water-salinity gradient.

**Figure 4.**
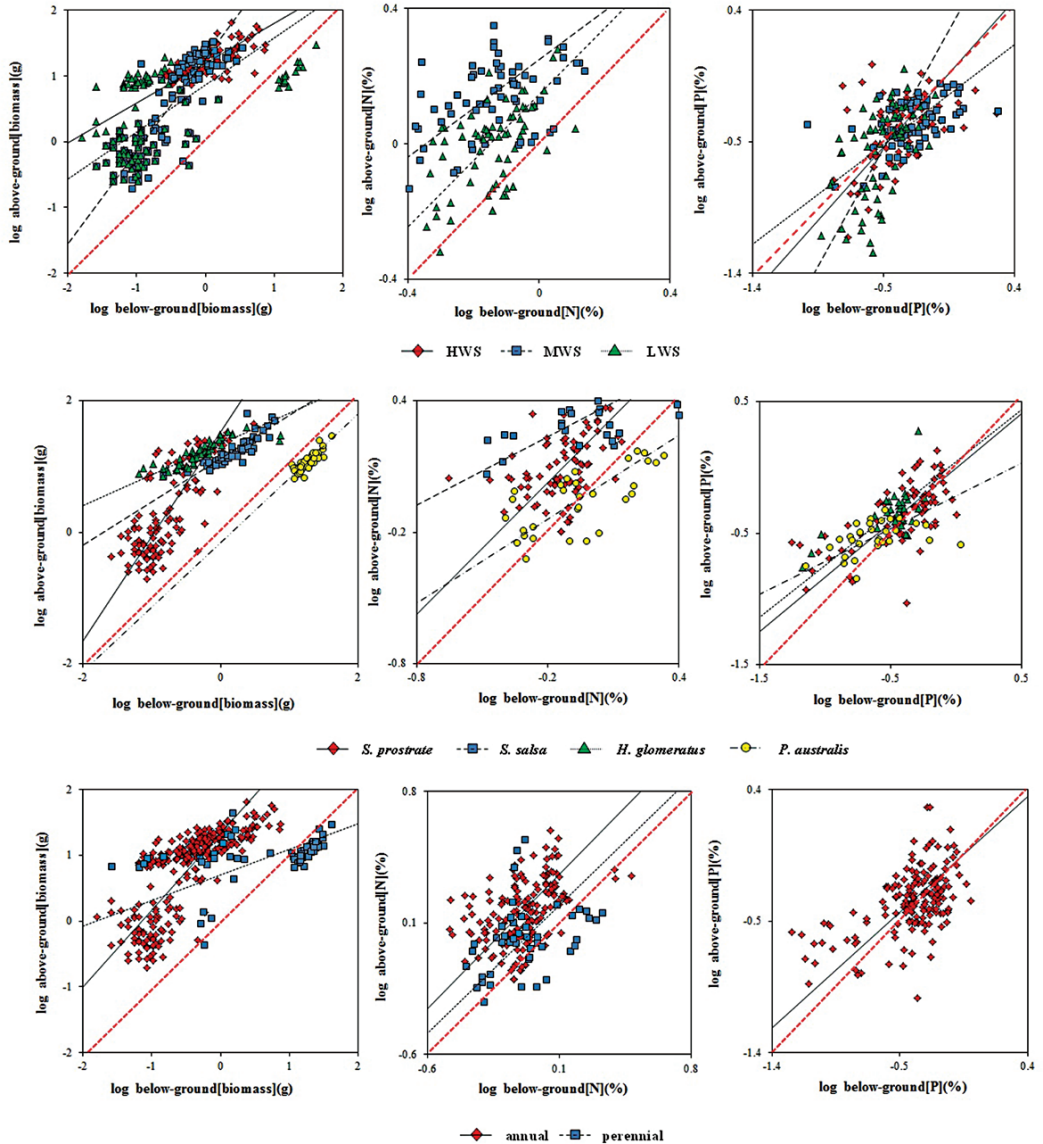
Scatterplots showed the RMA regressions of above- and below-ground biomass with N or P. For the water-salinity gradient: rhomboids and single lines, squares and dash lines, triangles and point lines, respectively, represent high (HWS), medium (MWS) and low (HWS) water-salinity plots; for species: rhomboids and single lines, squares and dash lines, triangles and point lines, circles and dotted lines, respectively, represent *S. prostrat*a, *S. salsa*, H. glomeratus and *P. australis*; for functional groups: rhomboids and single lines, squares and dash lines, respectively, represent annual and perennial plants. All black lines were significant RMA regressions (*P* < 0.05), and red dashed lines represent the slope equal to 1. All data were log_10_-transformed.

Both biomass and the concentrations of N and P showed significant correlations above- and below-ground at the water-salinity gradient, species, and functional group levels (Table 4; Fig. 4). At the gradient level, *α*_biomass_ was always significantly different from 1 (*P* < 0.001), showing an allometric relationship existed. In addition, *α*_N_ was similar to 1 at LWS (*P* < 0.01), showing an isometric relationship. The difference of *α*_P_ among gradients indicated that the lower the soil water and salinity were, the more obvious the allometric growth was.

**Table 4.** Summary of reduced major axis (RMA) regression results above- and below-ground.

	Biomass			N			P			
	α_biomass_	95%CI	*r* ^*2*^	α_N_	95%CI	*r* ^*2*^	α_P_	95%CI	*r* ^*2*^	*n*
HWS	**0.61** ***	0.52–0.71	0.50	**0.00**	−0.29–0.30	0.02	1.10***	0.9–1.4	0.20	120
MWS	**1.41** ***	1.29–1.54	0.71	**0.72** **	0.58–0.91	0.33	**0.80** ***	0.6–0.9	0.30	115
LWS	**0.72** ***	0.62–0.85	0.25	0.98**	0.79–1.23	0.15	**1.80** ***	1.5–2.2	0.30	120
*S. prostrata*	1.59***	1.43–1.77	0.63	1.00***	0.81–1.23	0.17	**0.83** ***	0.70–0.98	0.44	130
*S. salsa*	**0.66** ***	0.57–0.76	0.74	**0.52** *	0.37–0.72	0.23	**0.00**	−1.5–0.36	0.03	50
*H. glomeratus*	**0.47** ***	0.40–0.55	0.73	**0.00**	−0.07–0.56	0.08	0.79***	0.59–1.06	0.41	50
*P. australis*	0.98***	0.84–1.15	0.71	**0.64** ***	0.49–0.85	0.48	**0.50** *	0.36–0.71	0.19	50
Annual	**1.17** ***	1.09–1.27	0.59	1.02***	0.90–1.18	0.19	0.88***	0.78–1.01	0.30	275
Perennial	**0.39** ***	0.32–0.48	0.17	0.97*	0.73–1.29	0.12	**0.00**	-.07–0.56	0.05	75

***< 0.001;

**< 0.01;

*< 0.05; CI, confidence interval; *n*, sample size. Regression slopes (α _biomass_, α _N_ and α _P_) estimated in bold were significantly different from 1, indicating that the scaling relationship of the two traits are anisometric. HWS, MWS, and LWS represent high, medium, and low water-salinity plots, respectively.

An *α* above 1 indicated that above-ground increased linearly faster than below-ground; whereas an *α* below 1 indicated the increase of below-ground was faster than above-ground (Wright *et al*. 2004; Kerkhoff *et al*. 2006). Therefore, at HWS and LWS, the growth of below-ground biomass was faster than the above-ground which tends to increase at MWS. The growth of P was the fastest at LWS.

#### Scaling exponents across species.

There were significant scaling relationships between the above-ground biomass (N or P) along with the below-ground biomass and stoichiometry of four dominant species, but their scaling exponents varied among species (Table 4; Fig. 4). Except for *P. australis*, the other *α*_biomass_ measurements were significantly different from 1 (*P* < 0.001), showing an allometric relationship. In addition, *α*_N_ of *S. prostrata* was approximately 1, showing an isometric relationship (*P* < 0.001), while the measurements of *α*_N_ of *S. salsa* and *P. australis* were not significantly different from 1. Also, *α*_P_ of *S. prostrata* was similar to the isometric scaling (*P* < 0.001), and *α*_P_ of *P. australis* was significantly different from 1. All scaling exponents of *S. prostrata* were larger than other species, with *α*_biomass_ above 1 and bias towards elevated allocation to above-ground biomass.

#### Scaling exponents across functional groups.

Both the biomass and N concentration of annual and perennial plants had significant scaling relationships between above- and below-ground (*P* < 0.001, *P* < 0.05, respectively). The *α*_biomass_ of perennial plants was significantly less than 1 (*P* < 0.001), indicating that below-ground biomass grew faster than above-ground biomass. The *α*_N_ values of annual and perennial plants were similar to those of the isometric relationship (*P* < 0.05) (Table 4; Fig. 4).

### Patterns of scaling relationships in different scales

**Figure 5.**
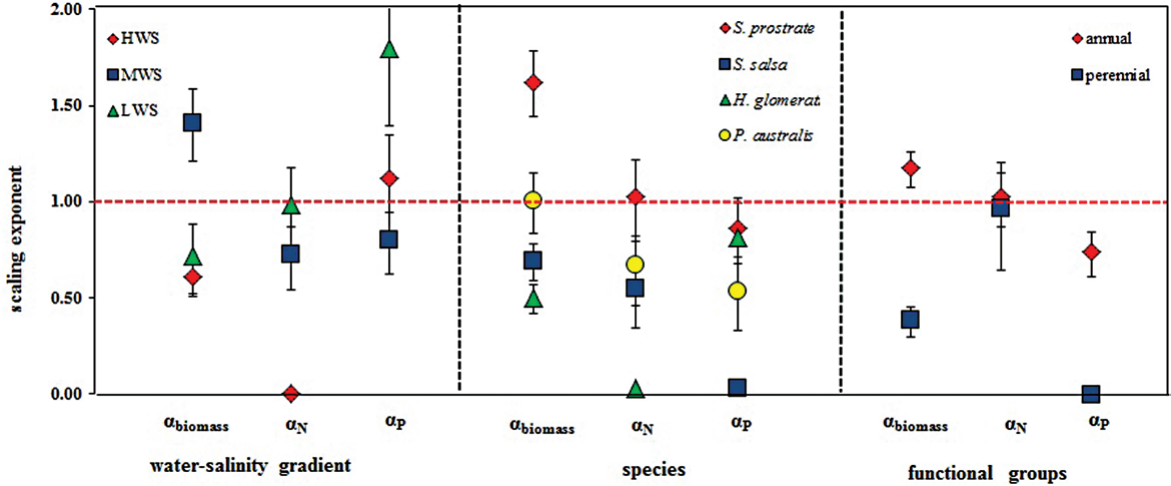
Comparisons of the scaling exponents *α*_biomass_, *α*_N_ and *α*_P_ among water-salinity gradient, species and functional group level. The error bars show the 95 % confidence interval (CI). The horizontal dashed line shows a slope value of 1 for reference. Note: HWS, MWS and LWS represent high, medium and low water-salinity plots, respectively.

At the water-salinity gradient level, the scaling exponents of the biomass were highest in MWS (1.41) followed by LWS and HWS (0.72, 0.61); and *α*_P_ measurements in MWS were smaller than those of LWS and HWS. The *α*_biomass_ of MWS was greater than 1, with *α*_N_ and *α*_P_ were less than 1; meanwhile, the amounts of *α*_biomass_ in LWS and HWS were less than 1, with *α*_N_ and *α*_P_ larger than or similar to 1. At the species level, the *α*_biomass_ of *S. prostrata* was the highest (1.59), followed by *P. australis* (0.98); also, the *α*_N_ and *α*_P_ of *S. prostrata* were higher than those of the other species but less than the *α*_biomass_. All scaling exponents of biomass along with N and P concentration of *S. salsa* and *H. glomeratus* were less than 1. Similar to *S. prostrata*, the *α*_biomass_ of *P. australis* was higher than *α*_N_ and *α*_P_, with the lowest of *α*_P_. From the functional group perspective, the *α*_biomass_ of annual plants (1.17) was significantly higher than that of perennial plants (0.39). The *α*_N_ and *α*_P_ of annual plants were similar among the functional groups (1.02 vs. 0.88), showing an isometric relationship (Table 4; Fig. 5).

## Discussion

### The influencing factors of above- and below-ground biomass with N, P contents of herbaceous plants

As the lowest elevation at the south-western margin of the Junggar Basin, the study area is the convergence centre of water and salt. Infiltration near the riverbank supplies the soil with moisture, while high evaporation causes high levels of salinity in the topsoil. The result is that soils with a relatively high water content also have a relatively high soil salinity (Qin *et al*. 2011), thereby coupling the spatial distribution pattern of soil water and salt, which provides microhabitats with substantial variations for the growth of plants. Therefore, plants regulate morphology (Geng *et al*. 2014) and stoichiometric traits (Zhang *et al*. 2012) with response to spatial resource heterogeneity; that is, the variation of species and functionality with water-salinity gradients are directly reflected in the patterns of plant biomass as well as N and P concentrations. Water-salinity gradients, species, and functional groups have various relative effects on the biomass and stoichiometry above- and below-ground. Through the strong individual and interactive effects, water-salinity gradients and species intensively influence biomass as well as N or P concentrations; in contrast, functional groups have no significant effect on the traits of plants (Table 2). Previous studies have demonstrated that the variations in the chemical traits of leaves (Ågren and Weih 2012; Zhang *et al*. 2012) and roots (Burton *et al*. 2000; Holdaway *et al*. 2011) are directly associated with the environmental conditions, including soil nutrient availability (Burton *et al*. 2000) and climate (Chen *et al*. 2013c). Conversely, stems with a high tissue density and slow metabolic activity will invest more in biomass costs and less in nutrient concentrations (Li *et al*. 2010; Fortunel *et al*. 2012). Zhao *et al*. (2016) found that the total effects of the plant functional group (trees, shrubs and herbs) on the N and P concentrations are associated with stems, compared with leaves and fine roots, and are relatively important for construction costs. The above-ground parts of herbaceous species in this study lack a strong supporting structure; thus, the functional groups (annual and perennial) are not responsible for the changes of the plant traits. However, the effects of water-salinity gradients and species suggest that the plant biomass and stoichiometry reflect both the changes in the micro-environment and plant intrinsic characteristics.

### Stoichiometric response of plants to soil on the water-salinity gradient

Here, the interaction of the plant N and P concentrations with soil nutrient availability were analysed using the water-salinity gradient (Fig. 3). The soil N concentrations were correlated positively with the above-ground N concentrations of *S. prostrata*, and negatively with the below-ground concentrations, which indicate that *S. prostrata* tends to allocate more N above-ground relative to below-ground. The soil P concentrations were positively correlated both above- and below-ground, which suggests that the P concentrations in the above- and below-ground organs change in a coordinated fashion. The difference may be caused by the properties of N and P under drought stress—plants under drought conditions usually increase their leaf N concentration to compensate for the low photosynthetic rate caused by a decrease in stomatal conductance (Wright *et al*. 2003; Palmroth *et al*. 2013). Unlike soil N, P in soil is derived from rock weathering; P has a lower dispersal ability in the soil solution than N (Lambers *et al*. 1998). The interaction effects of the soil nutrients and water-salinity gradients are more significant for plant P (*P* < 0.001), indicating that P concentrations in plant tissues are intensively influenced by soil water and P availability (Ordoñez *et al*. 2009; Wu *et al*. 2012; Hong 2016). Water and nutrients are known to restrict the productivity of plants in arid areas (Rao and Allen 2010), which further results in the co-limitation of N and P (He *et al*. 2008). The results of the present study also showed that the N and P concentrations of plants at HWS were higher than those at LWS (N: 2.01 vs. 1.01 %; P: 0.65 vs. 0.36 % for the mean value of above-ground). This suggests that, with a decrease in soil moisture, plants would reduce the nutrient content of the above- and below-ground organs in a coordinated way under stress condition, so as to maximize the adaptability of plants (Zhao *et al*. 2016).

### The differences in the allometric scaling of biomass with N, P contents on the water-salinity gradient

**Figure 6.**
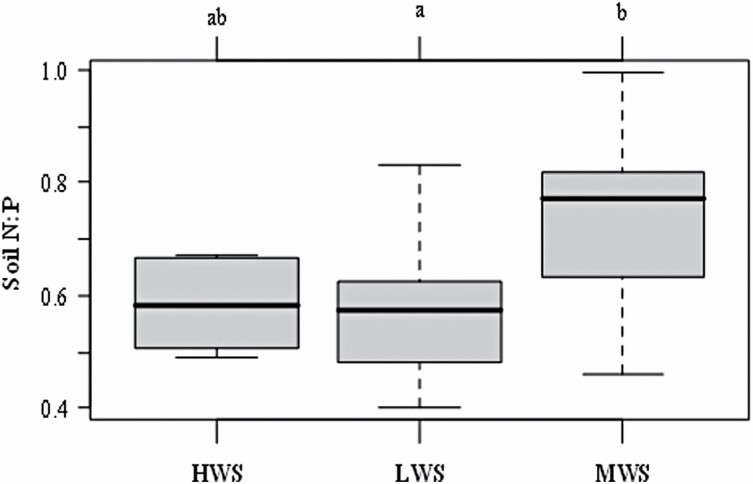
Soil N:P ratio in relation to water-salinity gradient. The lines and error bars show the means and standard errors for each gradient, respectively. The letters represent the significant difference among gradient. Note: HWS, MWS and LWS represent high, medium and low water-salinity plots, respectively.

In the present study, changes in the soil nutrients and properties resulted in changes in the plant traits. The scaling relationship of the biomass as well as N and P were different between the above- and below-ground parts, which vary with the water-salinity gradients. From the whole plant perspective, more biomass would be allocated to the root when plant growth is restricted by water, increasing the proportion of the below-ground biomass to promote the ability of the plant to acquire water (Hui and Jackson 2006; Mokany *et al*. 2006; Fan *et al*. 2009). However, *α*_biomass_ > 1 at MWS and <1 at HWS and LWS, indicating that plants tend to increase the above-ground biomass at MWS. With the exception of water, soil fertility is another factor affecting biomass allocation, and plants in infertile conditions tend to allocate more resources to roots rather than to the above-ground parts (Gleeson and Tilman 1990; Shipley and Meziane 2002; Robinson *et al*. 2010). For another, the growth of the root is usually considered to be more sensitive to P limitation than the crown of the plant (Güsewell 2005; Venterink and Güsewell 2010); the root growth and development will be more severely suppressed under a higher N:P ratio condition (P limitation), which contributes to the allometric relationship between the above- and below-ground parts (Luo *et al*. 2016). Soil N:P was highest at MWS where the roots are suppressed, so that the plants at MWS tend to have an allometric relationship (Fig. 6; **see****Supporting Information—Appendix B**).

With a decrease in the soil water and salinity, the allometric scaling of the N concentrations between the above- and below-ground parts gradually tended to be isometric, while the scaling relationships of P concentrations tended to a shift from isometric to allometric (0.00, 0.72 and 0.98 for *α*_N_; 1.10, 0.80 and 1.80 for *α*_P_; for the high, medium and low water-salinity point plots, respectively; Fig. 5). This suggests that the nutrient allocation tends to be above-ground with an increase in water and salt stress, and the N allocation strategy tends to be consistent; P has more plasticity to change its allocation strategies. The water shortage in the study area restricts the processes of soil mineralization and organification to slow down the release of P and limit N availability (Drenovsky and Richards 2004). Plants have the ability to coordinate the absorption and allocation of limited resources across various organs and to adapt to environmental constraints (Liu 2010). Plants respond to drought with a smaller leaf biomass and higher N and P concentrations (Wright *et al*. 2001; Wei *et al*. 2011); that is, the plants in arid areas can store more nutrients and use water more effectively and efficiently than other plants (Chapin *et al*. 1990; Wei *et al*. 2011). Generally, the changes in external environmental factors are coupled with the stoichiometric homeostasis of plants (Saterner and Elser 2002; Castle and Neff 2009); however, P is more variable in plant tissue than N (Yu *et al*. 2010). Existing evidence shows that certain positive correlations in N and P concentrations exists among the leaf, stem and root tissues (Kerkhoff *et al*. 2006; Geng *et al*. 2014). However, the coupling of stoichiometric traits could be the plasticity needed to vary with environmental change; that is, the scaling relationship of nutrients above- and below-ground is more variable relative to the soil properties.

### The interspecific differences in the allometric scaling of plant biomass and stoichiometric traits

Because different species have specific adaptations to different environments (Müller *et al*. 2000), allometric scaling is relatively conservative within a particular species (McCarthy and Enquist 2007). Interspecific variations exist in the scaling relationship of biomass and stoichiometry between the above- and below-ground parts. The scaling relationships tend to be allometric for the biomass of *S. prostrata* and isometric for the N and P concentrations (Table 4; Fig. 5), which are greater than those of other species; the scaling relationships tend to be isometric for biomass of *P. australis* and allometric for N and P concentrations; in addition, the overall effects on S. salsa and *H. glomeratus* biomass tend to be allometric, and all scaling exponents were less than 1. The optimal allocation theory suggests that plants would allocate more resources to organs that capture the greatest amounts limited resources (Poorter and Nagel 2000; Niklas and Enquist 2002). Limited water availability will result in an increase in the proportion of below-ground biomass to help plants absorb and store more water under drought stress (Hong *et al*. 2016). In addition to water and nutrients, the osmotic stress of saline soil contributes to a decrease in the amount of water available to plants (Bresler *et al*. 1982); osmotic stress will also suppress soil microbial activity, such as ammonification and nitrification, so as to slow down the N cycle of soil-plant (MoClung and Frankenber 1985). If extra costs of investment do not substantially increase resource acquisition, this may restrict the expression of the relationships among organs (Craine *et al*. 2005). In theory, a fundamental trade-off exists between the maximization of water acquisition and nutrient availability in infertile environments (Yan *et al*. 2016). In other words, plants allocate more biomass below-ground under arid conditions, but allocate more N above-ground to improve the photosynthetic capacity per unit of transpiring area (Field and Mooney 1986; Ye *et al*. 2015). All scaling exponents of *S. prostrata* were larger than those of other species, and this species tends to allocate more biomass and nutrients above-ground. That is, it uses a tolerance allocation strategy; the allometric scaling of N and P in *P. australis* may be derived from the greater nutrient acquisition capacity of grasses when compared to forbs (Luo *et al*. 2016); in addition, other species tend to increase the below-ground biomass along with the N and P concentrations, i.e. a ‘capture’ strategy. The interspecific variation in the allocation strategy drives species replacement on a micro-environmental gradient (Geng *et al*. 2014). The allometric pattern of annual plant species also expresses the similar adaptive strategy in that N and P concentrations tend to be isometric, and the biomass tends to be allometric.

## Conclusions

Our results suggest that biomass and stoichiometric traits of plants also vary with the micro-environment, not only with the scale of climate gradient. More importantly, there are some patterns whether nutrients of plants or the scaling relationships between the above- and below-ground, which show the trend of change with the water-salinity gradient. Although there is no more evidence to prove the mechanism, the hypothesis (2) has been confirmed. Finally, the interspecies difference of scaling relationships indicates that biomass and stoichiometric traits of plants reflect the trade-off in allocation strategies. Here, we describe as ‘tolerance’ or ‘capture’ strategy. Unfortunately, we only show the difference of scaling relationships of functional groups, but not the pattern, which requires more additional study to verify.

## Supporting Information

The following additional information is available in the online version of this article—

**Figure A1**. Bayesian estimation of parameters on HWS (high soil water and salinity) proves the feasibility of αN = 0.

**Figure A2**. Bayesian estimation of parameters on Suaeda salsa proves the feasibility of αN = 0.

**Figure A3**. Bayesian estimation of parameters on Halogeton glomeratus proves the feasibility of αN = 0.

**Figure A4**. Bayesian estimation of parameters on perennial proves the feasibility of αP = 0.

**Figure B1**. The soil N, P concentration and N:P ratio were distributed along the plots.

plab030_suppl_Supplementary_Appendix

plab030_suppl_Supplementary_Data

## Contributions by the Authors

G.H.L.: Conceptualization, Validation, Supervision; A.L.: Methodology, software, Writing-Original Draft; Y.Z.: Investigation, Data Curation; Z.F.H.: Investigation, Data Curation

## Data Availability

The data are available as **Supplementary Material**.

## Notes

**Plants, Ecosystems & Climate. Chief Editor:** Mary Heskel
