# Supplementary material for: Allometric scaling of biomass with nitrogen and phosphorus above- and below-ground in herbaceous plants varies along water-salinity gradients
Source: AoB Plants. 2021 Jun 9;13(4):plab030. doi: 10.1093/aobpla/plab030 (PMC8500215; doi:10.1093/aobpla/plab030)
Supplement: plab030_suppl_Supplementary_Appendix [file plab030_suppl_supplementary_appendix.pdf]

## Appendix A.

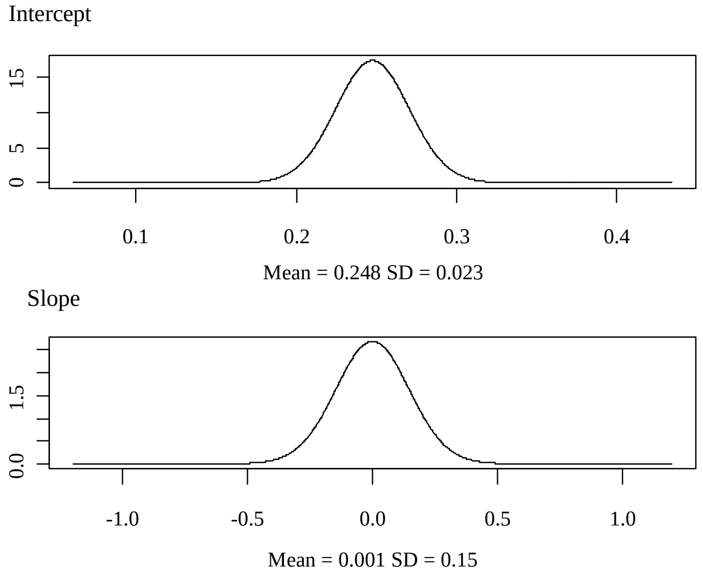


**Figure A1. Bayesian estimation of parameters on HWS (high soil water and salinity) proves the feasibility of $\alpha_N$ = 0.**

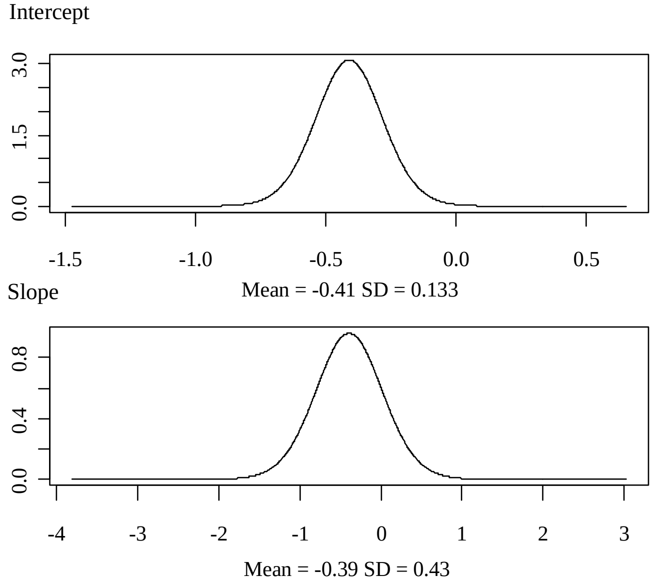


**Figure A2. Bayesian estimation of parameters on *Suaeda salsa* proves the feasibility of $\alpha_N$ = 0.**

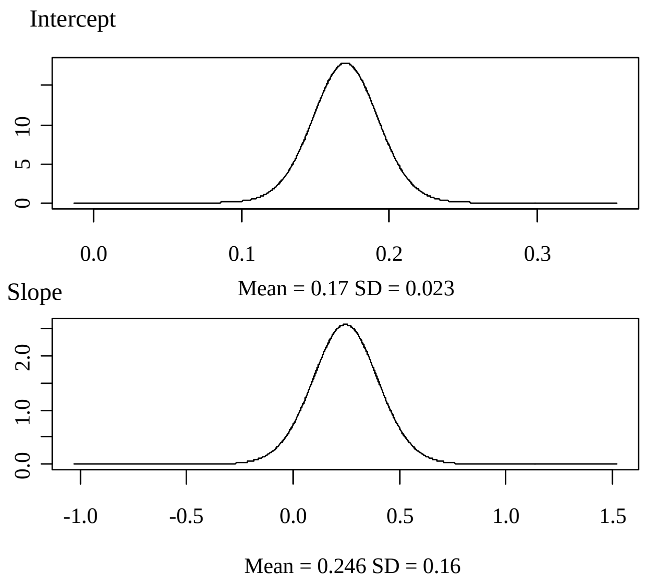


**Figure A3. Bayesian estimation of parameters on *Halogeton glomeratus* proves the feasibility of $\alpha_N = 0$.**

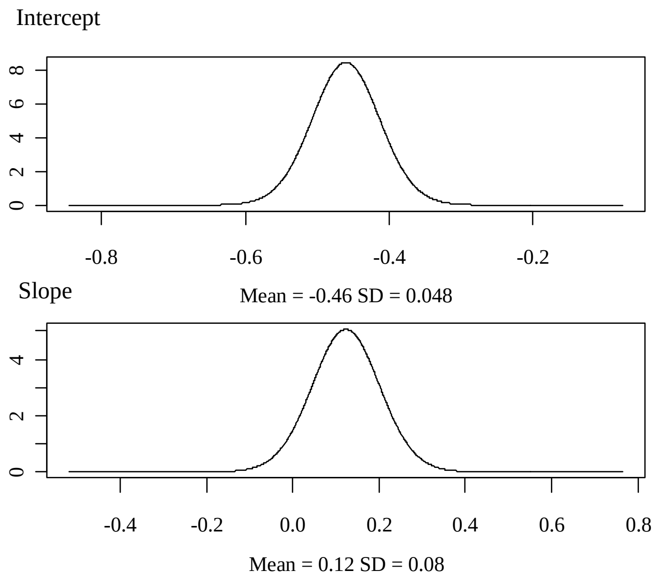


**Figure A4. Bayesian estimation of parameters on perennial proves the feasibility of $\alpha_P = 0$.**

**Appendix B.**

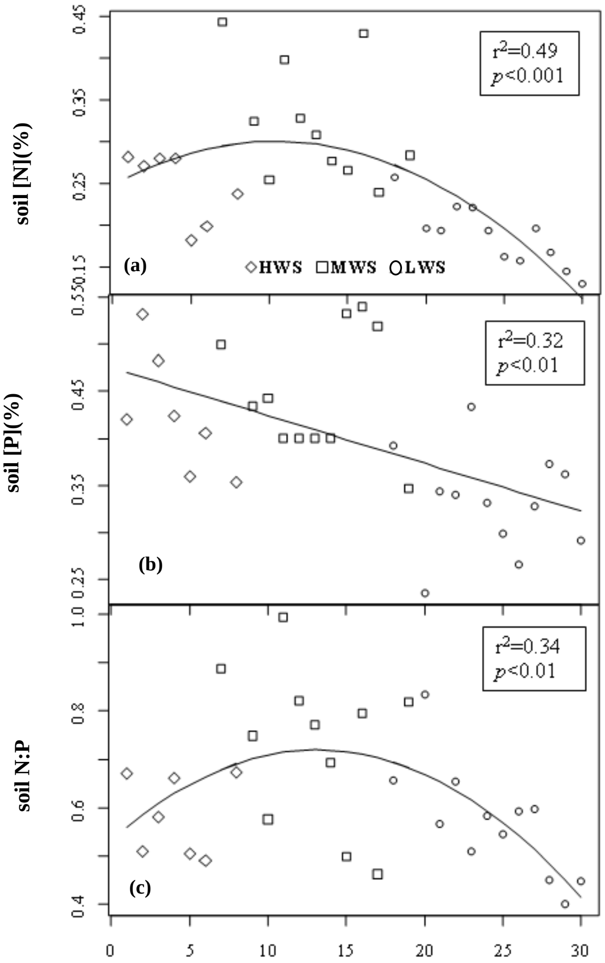


**Figure B1. The soil N, P concentration and N:P ratio were distributed along the plots.** The rhomboids represented HWS, the squares represented MWS, and the triangles represented LWS. Regression $r^2$ values were given in the figure respectively.
